# Floral visitors and potential pollinators of *Calanthe discolor* and *C. striata* in South Korea

**DOI:** 10.3897/BDJ.14.e201540

**Published:** 2026-08-24

**Authors:** Gyu Young Han, Seung-Su Euo, Sealki Son, Heung-Sik Lee, Kyung Choi, Chang-Jun Kim

**Affiliations:** 1 Forest Biodiversity Research Division, Korea National Arboretum, Pocheon, Republic of Korea Forest Biodiversity Research Division, Korea National Arboretum Pocheon Republic of Korea; 2 Department of Ecological Science, Kyungpook National University, Sangju, Republic of Korea Department of Ecological Science, Kyungpook National University Sangju Republic of Korea; 3 Department of Forest Environment Protection, Kangwon National University, Chuncheon, Republic of Korea Department of Forest Environment Protection, Kangwon National University Chuncheon Republic of Korea; 4 Department of Plant Pest Control, Animal and Plant Quarantine Agency, Gimcheon, Republic of Korea Department of Plant Pest Control, Animal and Plant Quarantine Agency Gimcheon Republic of Korea

**Keywords:** *Calanthe discolor*, *Calanthe striata*, Orchidaceae, potential pollinators, pollinaria transport

## Abstract

Numerous *Calanthe* (Orchidaceae) species are considered food-deceptive orchids because they attract floral visitors without providing rewards. In this study, we investigated floral visitors and their different types of behaviour in natural populations of *Calanthe discolor* and *C. striata* in South Korea during peak flowering to identify pollinaria-carrying visitors and assess whether the observations suggest potential pollinator sharing between the two taxa. A total of 73 visitation events involving 17 insect species were recorded on *C. discolor*. Diptera accounted for most visitation events, but no dipteran or lepidopteran visitors carried pollinaria. Pollinaria attachment was confirmed only in hymenopteran visitors (*Andrena opacifovea*, *Apis cerana*, *Ceratina japonica* and *Lasioglossum formosae*) that probed deeply into the spur. In *C. striata*, a single visitation event by *Eucera nipponensis* was recorded and the observed individual carried pollinaria on the head and transported them between flowers. This observation is consistent with previous reports from Japan and suggests a possible mechanism of pollinator sharing that should be tested through broader temporal and population-level observations. The results reveal a clear mismatch between visitation frequency and pollinaria transport in *C. discolor*, with pollinaria attachment restricted to a subset of bee visitors. Together with previous studies, our findings support a generalised food-deceptive pollination strategy in *C. discolor* mediated by locally available bees. This study provides baseline field evidence on floral visitors and pollinaria transport in Korean *Calanthe* and establishes testable hypotheses for future studies of reproductive interactions amongst closely-related taxa.

## Introduction

Orchid reproduction often depends on specialised interactions with floral visitors, making pollinator identity a key determinant of fruit set and population persistence (Johnson and Steiner 2000, Cozzolino and Widmer 2005). These interactions are mediated by diverse floral traits and reward strategies, ranging from nectar- and pollen-rewarding flowers to deceptive systems that offer little or no reward (Tremblay 1992, Sugiura 2013, Ren et al. 2014). Specifically, in deceptive orchids, visitor behaviour — how visitors approach, handle and depart from flowers — can strongly influence the likelihood of pollinaria removal and subsequent deposition (Tremblay 1992, Ren et al. 2014). Such fine-scale variation in plant–pollinator interactions can shape reproductive success and may contribute to ecological divergence and diversification across orchid lineages (Johnson and Steiner 2000, Cozzolino and Widmer 2005). Most *Calanthe* species employ a food-deceptive strategy, which typically results in lower visitation frequencies and fruit set than in rewarding plants (Sugiura 2013, Ren et al. 2014, Sakata et al. 2014). The genus comprises about 200 species of perennial terrestrial orchids distributed primarily across Africa, Madagascar, China, Japan, tropical Asia and Australia (Suetsugu and Fukushima 2014, Suetsugu and Tanaka 2020). To date, only approximately eight species within the genus have been reported as autogamous, with the majority relying on cross-pollination (Jacquemyn et al. 2005, Zhang et al. 2024). Previous studies in Japan and China have identified medium- to large-sized bees (*Xylocopa*, *Eucera*, *Bombus*) and sometimes sweat bees (*Lasioglossum*) as major pollinators (Sugiura 2013, Ren et al. 2014, Sakata et al. 2014, Suetsugu and Fukushima 2014, Suetsugu et al. 2016). Additionally, hesperiid butterflies have been confirmed as effective pollinators of *Calanthe* species in south-eastern China (Luo et al. 2020). Owing to their high ornamental value, *Calanthe* species have been subject to overcollection and habitat destruction, contributing to declines in wild populations and placing some species (e.g. *C. amamiana*) at risk (Chung et al. 2013, Sugiura 2022). While artificial propagation techniques using ultrasonic or chemical treatments have been established to aid ex situ conservation, understanding their pollination ecology in the wild remains crucial (Miyoshi and Mii 1988, Miyoshi and Mii 1995, Kim et al. 2008).

In South Korea, seven *Calanthe* species have been recorded: *C. discolor*, *C. sieboldii*, *C. striata*, *C. reflexa*, *C. aristulifera*, *C. insularis* and *C. rubra* (Hyun et al. 1999, Hong et al. 2009, Hong et al. 2010, Chung et al. 2013, Oh et al. 2015). Although Plants of the World Online (POWO 2026) treats *C. sieboldii* as a synonym of *C. striata*, we recognise them as distinct taxa in the present study, following the taxonomic treatment adopted in the Korean Plant Names Index (KPNI; Korea National Arboretum (2025)) and the Korean floristic and conservation literature cited here. Amongst these, *C. aristulifera* is designated as a Class II Endangered Wildlife species (National Institute of Biological Resources 2023) and *C. reflexa* and *C. sieboldii* are classified as Endangered (EN) in the Korean National Red List (Korea National Arboretum 2021). Although a recent field-based pollination study has been reported for *C. aristulifera* in South Korea, field data on floral visitor assemblages and pollinaria transport in wild *Calanthe* populations in Korea remain limited (Kim et al. 2026).

These species are largely restricted to the understorey of evergreen broad-leaved forests on the southern coastal islands and Jeju Island, where a relatively warm oceanic climate prevails (Park et al. 2010, Kim et al. 2012). In Korean botanical literature, *C. striata* has been treated as a putative natural hybrid involving *C. discolor* and *C. sieboldii* and occurs sympatrically with these taxa on Jeju Island, where it exhibits extensive morphological variation (Hyun et al. 1999, Cho et al. 2009). Identifying floral visitors capable of removing and transporting pollinaria is an essential first step towards understanding reproductive interactions within this system, as hybridisation is a prevalent evolutionary process in orchids (Cozzolino et al. 2006, Nakahama et al. 2019). Genetic evidence indicates that *C. discolor* and *C. striata* are closely related, but the ecological mechanisms facilitating pollen movement amongst them in Korean populations remain unclear (Srikanth et al. 2013). In Japan, *Eucera nipponensis* has been reported as a pollinator of hybrids between *C. discolor* and *C. striata*, raising the possibility that closely-related *Calanthe* taxa may share pollinators (Suetsugu and Tanaka 2020). However, whether similar pollinaria-carrying visitor assemblages occur in Korean populations has not been examined using field observations.

Therefore, we investigated floral-visitor diversity and foraging behaviour in natural populations of *C. discolor* and *C. striata* in South Korea to document pollinaria-carrying visitors and evaluate whether the observations suggest a testable hypothesis of pollinator sharing between the two taxa. By documenting visitation events, visitor behaviour and pollinaria attachment, we provide baseline ecological information relevant to conservation and future studies of reproductive interactions amongst Korean *Calanthe* taxa.

## Materials and Methods

### Study species and survey period

**Figure 1.**
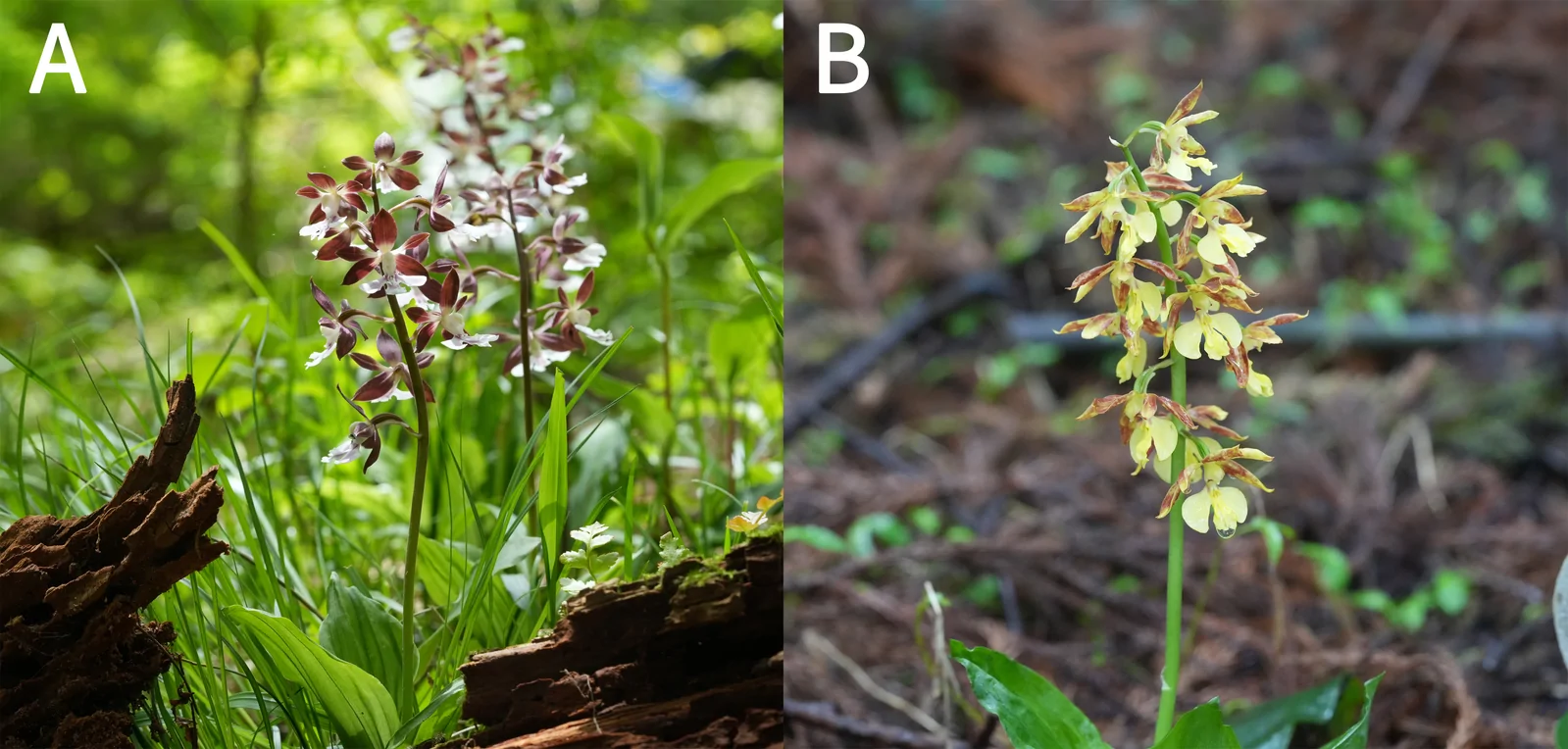
Flowers of *Calanthe* species. **A** *C. discolor*. **B** *C. striata*. Photos by S.S. Euo.

Field observations were conducted during the flowering season in natural populations of *Calanthe discolor* and *C. striata* in South Korea (Fig. 1). Surveys were synchronised with the peak blooming period, defined as the stage when more than 50% of the florets on the acropetal racemes were open. To maximise insect activity, field observations were conducted on clear, sunny days: 24 April 2025, for *C. striata* and 8 May 2025, for *C. discolor*.

### Study area

**Figure 2.**
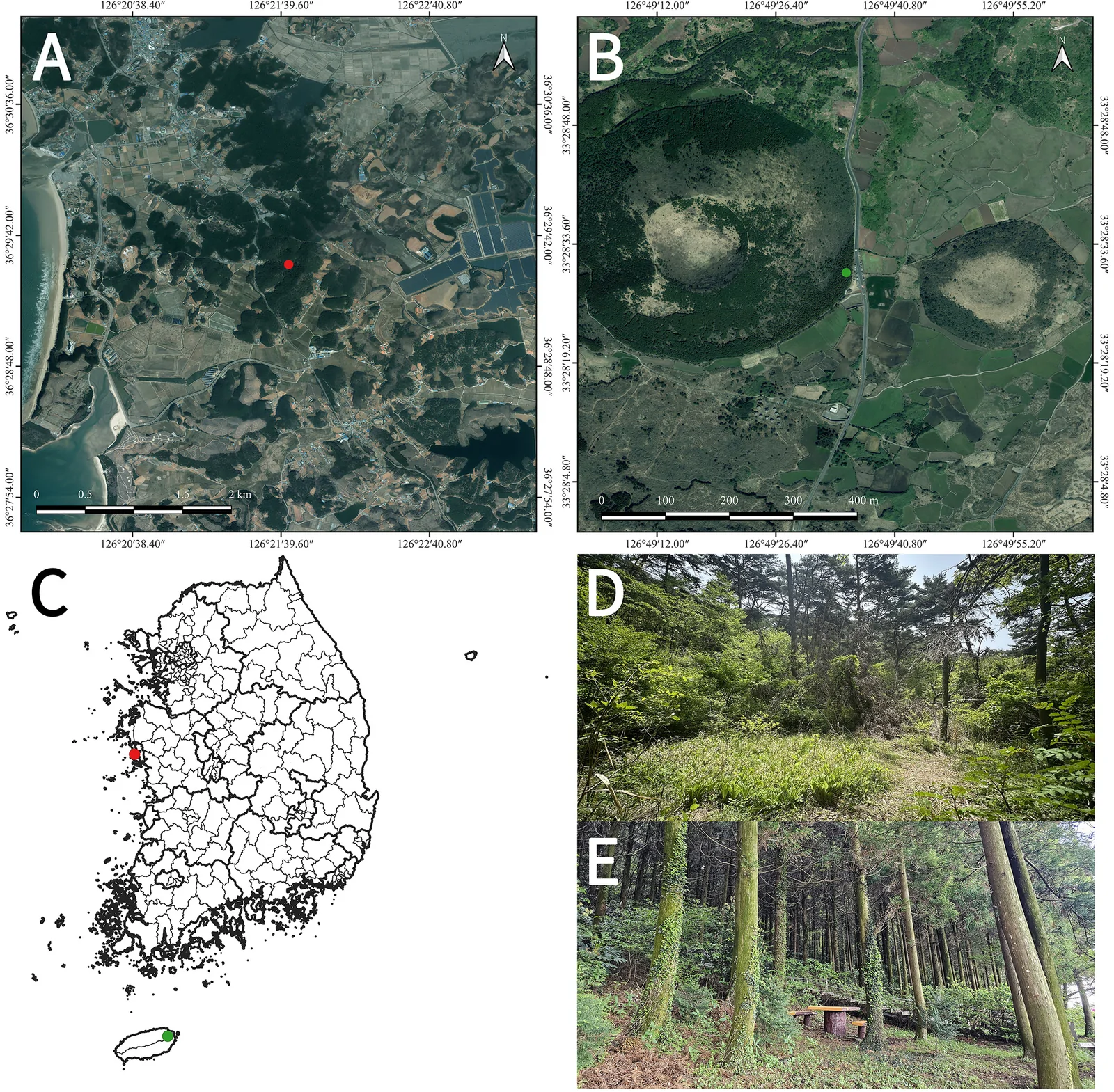
Survey sites and habitat conditions of *Calanthe discolor* and *C. striata* in South Korea (*C. discolor*, red dot; *C. striata*, green dot). **A** Satellite view of the *C. discolor* survey site on Anmyeon Island, Taean-gun, Chungcheongnam-do; **B** Satellite view of the *C. striata* survey site at Darangswi Oreum, Jeju-do; **C** Map of South Korea showing the locations of the two survey sites; **D** Natural habitat of *C. discolor* at Anmyeon Island; **E** Natural habitat of *C. striata* at Darangswi Oreum, Jeju-do. Habitat photographs in D and E by G.Y. Han and S.S. Euo.

Fieldwork was conducted in two habitats representing the natural ranges of the focal species: a population of *C. discolor* in the mixed forests of Anmyeon Island (Taean-gun, Chungcheongnam-do; 126°21'42.76"E, 36°29'30.02"N) and a population of *C. striata* in the dense Japanese cedar (*Cryptomeria japonica*) stands of Darangswi Oreum (Gujwa-eup, Jeju-si, Jeju-do; 126°49'34.93"E, 33°28'30.15"N) (Fig. 2). The Anmyeon Island site contained more than 100 individuals of *C. discolor* distributed across approximately 10 patches, of which 75 flowering individuals in three patches were included in the present observations. At the Darangswi Oreum site, approximately 10 flowering individuals of *C. striata* occurred in a single patch and all were included in the observations. Although the vegetation structure differed between the two sites, both were characterised by a dense canopy that allowed the formation of ephemeral sunflecks on the forest floor, creating a dynamic light environment. To account for these spatiotemporal variations in microclimate, observers moved between orchid patches during the survey to track insect activity effectively.

Meteorological data were obtained from the nearest Korea Meteorological Administration stations. The mean ambient temperature on the survey days was 15.1°C (range: 6.6–22.0°C) in Anmyeon Island and 14.7°C (range: 9.8–18.0°C) in Jeju.

### Field observations and assessment of pollinaria-carrying visitors

Floral visitation was monitored continuously for 12 h (09:00–21:00) at each site. A total of four trained observers recorded visitor behaviour and the time of each visitation event. Behavioural observation categories were adapted from Sugiura (2013) and included approach (approaching a flower without landing), alighting/resting (landing on the flower surface) and nectar seeking (probing behaviour associated with foraging attempts); pollinaria attachment was assessed through direct field observation.

A visitation event was defined as one continuous encounter between an insect and an inflorescence, beginning when the insect approached the inflorescence and ending when it left the immediate vicinity. Movements amongst flowers within the same inflorescence were treated as part of the same visitation event. As visitors were not individually marked and not all visitors were captured, the counts represent visitation events rather than unique insect individuals and may include repeated visitation events by the same individual.

To minimise disturbance to plants and ensure observation of the complete visitation sequence, insects were captured using targeted netting only upon departure from the inflorescence. All collected specimens were mounted and morphologically identified by specialists for each taxonomic group. Voucher specimens were deposited at the Korea National Arboretum (KNA; https://kna.forest.go.kr).

To distinguish floral visitors from pollinaria-carrying visitors, all visitors were examined in the field for the presence of attached pollinaria. For captured specimens, the presence or absence of pollinaria was subsequently re-examined under a stereomicroscope in the laboratory. Floral visitors were defined as insects involved in a recorded visitation event, whereas pollinaria-carrying visitors were those observed bearing one or more pollinaria on their bodies. For each pollinaria-carrying visitor, the specific attachment site of the pollinaria on the insect’s body was recorded.

## Data resources

The data underpinning the analysis reported in this paper are deposited at GBIF, the Global Biodiversity Information Facility, https://doi.org/10.15468/58ygbq.

##  Results

### Calanthe discolor: Visitor composition and pollinaria attachment

**Figure 3.**
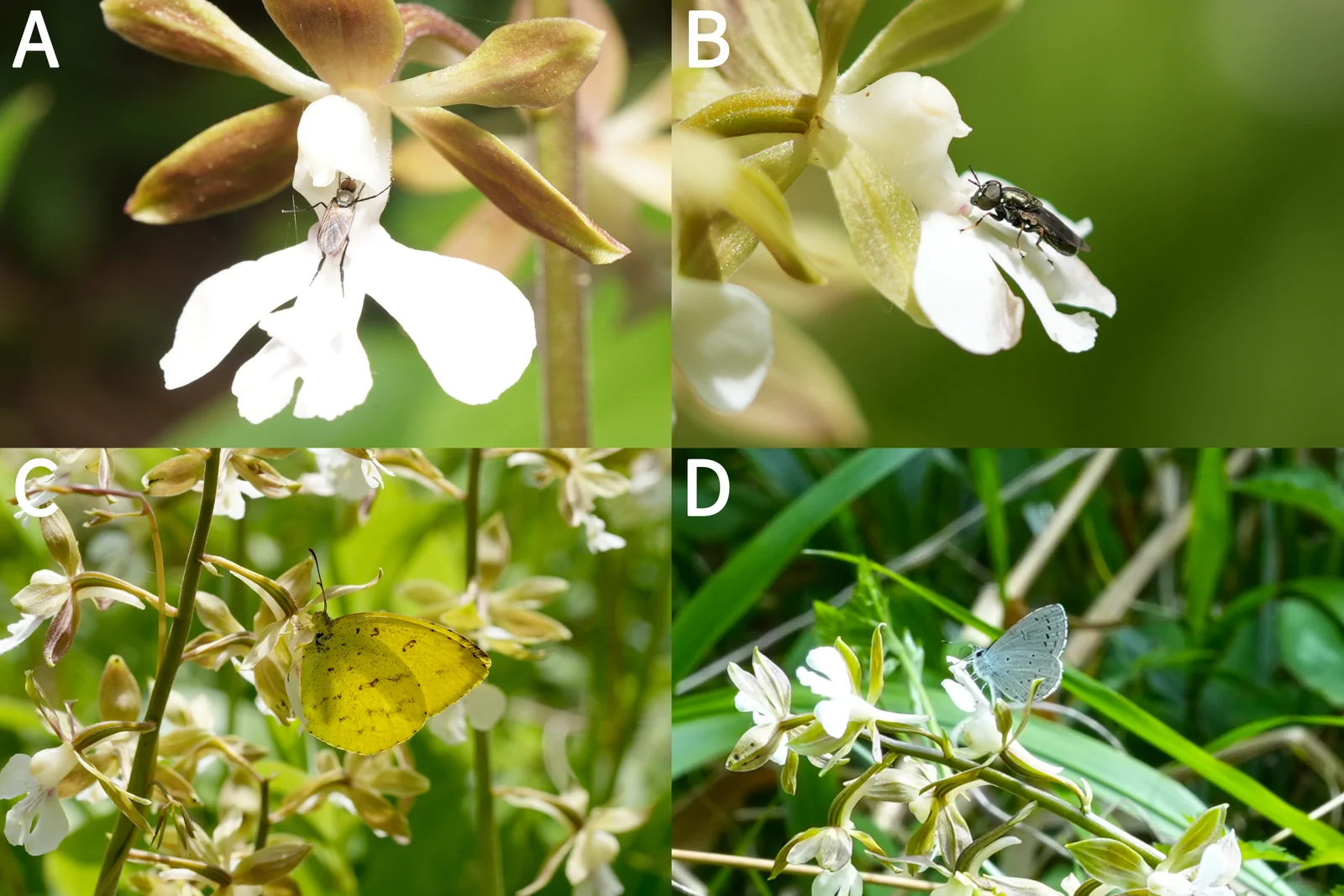
Floral visitors of *Calanthe discolor*. **A** *Empis* sp.; **B** *Orthonevra karumaiensis*; **C** *Celastrina argiolus*; **D** *Eurema mandarina*. Photos by G.Y. Han and S.S. Euo.

**Figure 4.**
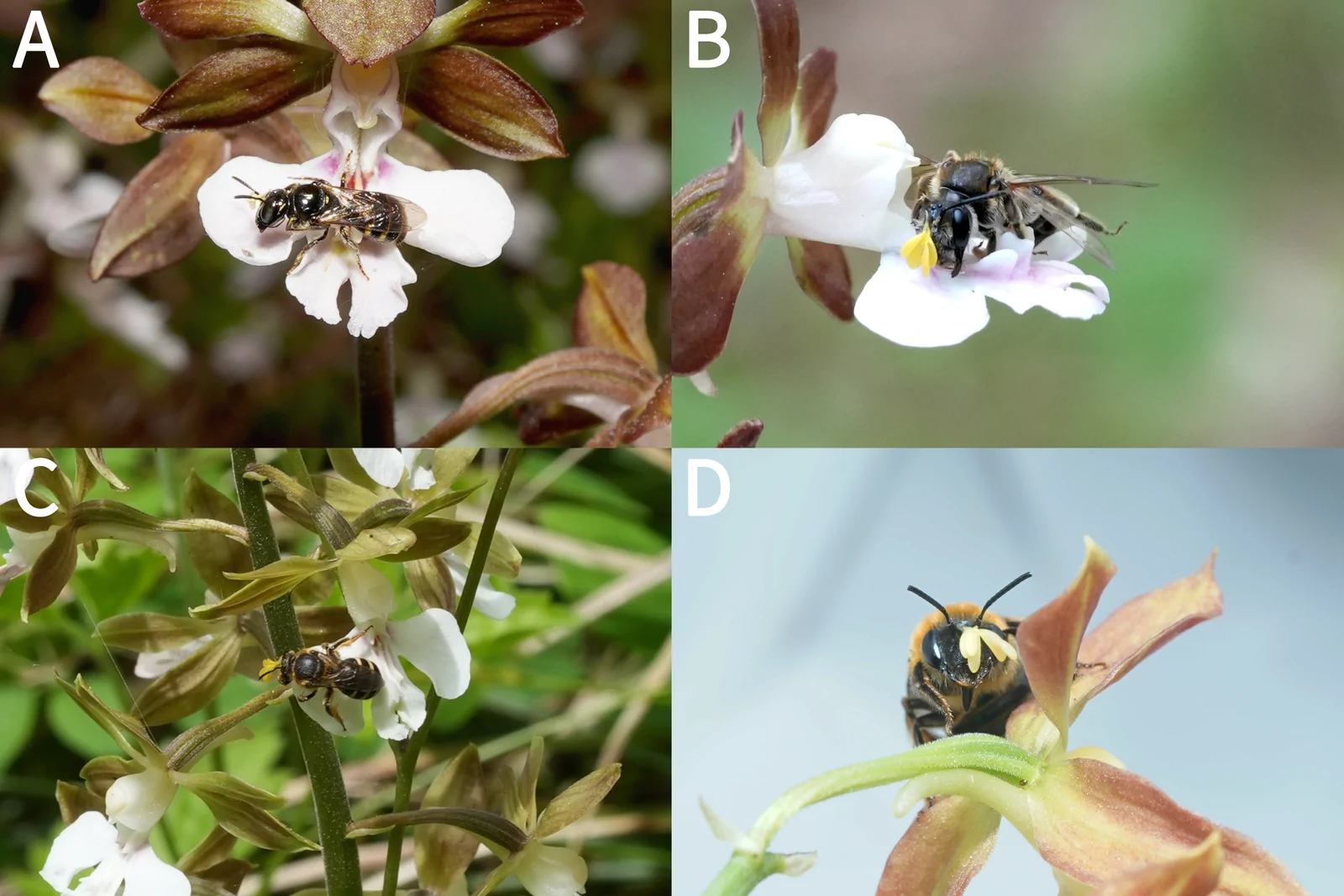
Pollinaria-carrying visitors of *Calanthe discolor* and *C. striata*. **A–C** *C. discolor*; **D** *C. striata*. **A** *Ceratina japonica*; **B** *Andrena opacifovea*; **C** *Lasioglossum formosae*; **D** *Eucera nipponensis*. Photos by G.Y. Han and S.S. Euo.

A total of 73 visitation events involving 17 species, nine families and three orders were recorded on *C. discolor* (Table 1). Visitation occurred only during daytime hours and no visitor activity was observed at night. Diptera accounted for most visitation events, mainly involving *Empis* sp. and five syrphid species (Fig. 3A and B). *Empis* sp. accounted for the largest number of visitation events (42 events) and was recorded throughout the daytime (Table 1). Tachinidae was represented by a single visitation event involving *Ectophasia rotundiventris*. Two lepidopteran taxa, *Celastrina argiolus* and *Eurema mandarina*, were also recorded during the daytime, but neither carried pollinaria (Fig. 3C and D; Table 1). Despite frequent approaches and alighting, dipterans typically showed surface lapping or other shallow foraging behaviour and none carried pollinaria. In contrast, pollinaria attachment was confirmed only in Hymenoptera — *Andrena opacifovea*, *Apis cerana*, *Ceratina japonica* and *Lasioglossum formosae* (Fig. 4A–C) — all of which probed deeply into the spur; these pollinaria-carrying bee visitors were recorded from late morning to afternoon (Table 1).

### Calanthe striata: Pollinaria transport

Only one visitation event by *Eucera nipponensis* was recorded on *C. striata* during the daytime survey period. The observed individual carried pollinaria attached to the head and transported them between flowers (Fig. 4D). This visitation event occurred at 11:14 h (Table 1).

## Discussion

### Behavioural mismatch between visitation and pollinaria transport

In our study, most visitation events to *Calanthe discolor* were made by Diptera, particularly Empididae. Despite frequent visitation, no dipteran visitor was found carrying pollinaria during the present survey. This likely reflects a mismatch between visitor behaviour and floral architecture: surface lapping and other shallow foraging behaviour may not have brought the flies into contact with the viscidium on the column, which is necessary for pollinaria attachment. In contrast, hymenopteran visitors showed deep probing into the spur, presumably in search of nectar. Pollinaria attachment was observed exclusively in hymenopterans (*Andrena*, *Apis*, *Ceratina* and *Lasioglossum*) and attachment occurred on the head region, consistent with deep probing behaviour (Nilsson 1992, Ackerman et al. 2023). These bees can, therefore, be regarded as potential pollinators of *C. discolor*, although their relative pollination effectiveness remains to be tested. Recent monitoring of *C. aristulifera* in South Korea similarly suggests that the presence or frequency of floral visitors should not necessarily be equated with successful pollination under pollinator-limited conditions (Kim et al. 2026). Likewise, our observations show that high visitation frequency in a deceptive orchid does not necessarily result in pollinaria attachment or transport.

### Generalised food-deceptive pollination strategy of Calanthe discolor

A previous Japanese study reported that *C. discolor* is mainly pollinated by *Apis cerana*, *Eucera nipponensis* and *Osmia cornifrons* (Suetsugu and Fukushima 2014). In contrast, our study at Anmyeon Island recorded *Andrena opacifovea*, *Ceratina japonica* and *Lasioglossum formosae*, alongside *A. cerana*, as pollinaria-carrying visitors. Twelve visitation events involving *C. japonica* were recorded, whereas *E. nipponensis*, which has been reported as a major pollinator in mainland Japan, was not observed on *C. discolor* during the present survey. This contrast is consistent with possible geographic or local variation in pollinaria-carrying visitor assemblages. However, the short survey period and unmeasured differences in patch structure, floral display, habitat conditions and the surrounding plant community prevent us from identifying the factors responsible for this difference. The involvement of relatively small bees, such as *C. japonica*, further suggests that the floral structure of *C. discolor* can accommodate pollinaria attachment across a range of bee body sizes. Such flexibility may permit pollinaria attachment by locally available bee taxa of different body sizes. In addition, reports of *Lasioglossum* as a pollinator of *C. aristulifera* in the Izu Islands (Suetsugu et al. 2016) suggest that this genus may function as a recurrent pollinator resource within *Calanthe*. At a broader regional scale, hesperiid butterflies have been reported as effective pollinators of *Calanthe* species in south-eastern China (Luo et al. 2020), whereas neither of the lepidopteran visitors recorded in the present survey carried pollinaria. This comparison further suggests that assemblages of potential pollinators may vary amongst *Calanthe* species and geographic regions. Collectively, our short-term observations, together with existing literature, support a generalised food-deceptive strategy in *C. discolor*, in which the species recruits locally available bees rather than relying on a single specialised pollinator (Johnson and Steiner 2000).

### Eucera nipponensis as a potential pollinator of Calanthe striata

A single visitation event by *Eucera nipponensis* was recorded on *C. striata* and the observed individual transported pollinaria attached to its head between flowers. Although based on a single observation, this record provides field evidence that *E. nipponensis* is capable of removing and transporting pollinaria of *C. striata* in Korea. *Calanthe sieboldii* is reported to be more strongly associated with carpenter bees (*Xylocopa* spp.) (Sugiura 2013), whereas *C. discolor* is pollinated by a broader assemblage of bees that includes *Eucera* (Suetsugu & Fukushima 2014). Our observation is also consistent with a previous Japanese report of *E. nipponensis* visiting closely-related *Calanthe* taxa (Suetsugu and Tanaka 2020). Together, these records suggest that *Eucera* may participate in the pollination of multiple closely-related *Calanthe* taxa in East Asia. However, because no *Eucera* visitation event was recorded on *C. discolor* during the present survey, pollinator sharing between the Korean populations remains a hypothesis requiring broader temporal and population-level investigation.

The present study provides baseline field evidence of a clear distinction between visitation frequency and pollinaria transport in *C. discolor*, identifies four bee species as potential pollinators of *C. discolor* and provides evidence that *E. nipponensis* is a potential pollinator of *C. striata* in Korea. These findings should nevertheless be interpreted in light of the limited temporal and spatial replication, with one 12-h survey conducted at a single study site for each taxon, the single visitation event recorded for *C. striata* and the absence of direct measurements of pollinia deposition and fruit set. Accordingly, the observed records represent a temporal and spatial snapshot rather than a complete characterisation of the floral visitor assemblages or the relative pollination effectiveness of the recorded visitors. Repeated surveys across flowering periods and populations, together with direct measurements of reproductive success and relevant floral and habitat characteristics, will help test the ecological patterns suggested by the present observations.

## Data Availability Statement

The raw visitation records supporting this study, including observation date, time, site, focal taxon, visitor identity, observed behaviour, pollinaria attachment, attachment site and voucher information, are provided in Suppl. material 1.

## Supplementary Material

13EC3C56-C86F-5E06-874B-4237B3C20A23 10.3897/BDJ.14.e201540.suppl1 Supplementary material 1 Raw records of floral visitors and potential pollinators of Calanthe discolor and C. striata in South Korea Data type Occurrence records Brief description Raw records of floral visitation events observed for *Calanthe discolor* and *C. striata* in South Korea, including observation date, time, locality, plant species, visitor identity, observed behaviour, pollinaria attachment and attachment site and voucher specimen information where available. File: oo_1717003.xlsx https://binary.pensoft.net/file/1717003 Gyu Young Han, Seung-Su Euo, Sealki Son, Heung-Sik Lee, Kyung Choi, Chang-Jun Kim

## Figures and Tables

**Table 1.** Visitation behaviour and pollinaria attachment recorded for floral visitors to *Calanthe discolor* and *C. striata*.

Flower	Order	Family	Species	Approach	Alighting/resting	Nectar seeking	Pollinaria attachment	No. of visitation events	Observation time
*C. discolor*	Hymenoptera	Andrenidae	Andrena opacifovea*		●	●	●	1	12:13
		Andrenidae	*Andrena sasaki*		●	●		1	12:45
		Andrenidae	*Andrena yamato*		●	●		1	13:10
		Apidae	*Apis cerana*		●	●	●	2	11:50, 11:52
		Apidae	Ceratina japonica*		●	●	●	12	11:36–14:54
		Apidae	*Nomada* sp.		●	●		2	13:53, 14:43
		Halictidae	Lasioglossum formosae*		●	●	●	1	12:59
		Megachilidae	*Osmia taurus*		●	●		1	11:43
	Diptera	Empididae	*Empis* sp.	●	●	●		42	Throughout daytime
		Syrphidae	*Episyrphus balteatus*	●		●		2	15:23, 16:40
		Syrphidae	*Eristalis kyokoae*	●				1	12:44
		Syrphidae	*Eupeodes corollae*		●			1	14:49
		Syrphidae	*Orthonevra karumaiensis*		●	●		1	13:50
		Syrphidae	*Paragus haemorrhous*	●	●	●		2	13:22, 14:31
		Tachinidae	*Ectophasia rotundiventris*		●			1	14:45
	Lepidoptera	Lycaenidae	*Celastrina argiolus*		●			1	16:18
		Pieridae	*Eurema mandarina*		●			1	15:30
*C. striata*	Hymenoptera	Apidae	*Eucera nipponensis*		●	●	●	1	11:14

Note: Filled circles indicate types of behaviour or pollinaria attachment observed at least once for each visitor taxon; blank cells indicate that the behaviour or pollinaria attachment was not observed. Values in the “No. of visitation events” column indicate the total number recorded for each taxon. Asterisks indicate species first reported here as pollinaria-carrying visitors of *C. discolor*.
